# A Review of Membrane-Based Biosensors for Pathogen Detection

**DOI:** 10.3390/s150614045

**Published:** 2015-06-15

**Authors:** Remko van den Hurk, Stephane Evoy

**Affiliations:** Department of Electrical and Computer Engineering, University of Alberta Edmonton, Alberta, AB T6G 2V4, Canada; E-Mail: remko@ualberta.ca

**Keywords:** biosensors, pathogens, membranes, bacteria, food safety, water safety

## Abstract

Biosensors are of increasing interest for the detection of bacterial pathogens in many applications such as human, animal and plant health, as well as food and water safety. Membranes and membrane-like structures have been integral part of several pathogen detection platforms. Such structures may serve as simple mechanical support, function as a part of the transduction mechanism, may be used to filter out or concentrate pathogens, and may be engineered to specifically house active proteins. This review focuses on membrane materials, their associated biosensing applications, chemical linking procedures, and transduction mechanisms. The sensitivity of membrane biosensors is discussed, and the state of the field is evaluated and summarized.

## 1. Introduction

Pathogenic bacteria cause extensive illnesses and mortality around the globe. Contaminated water and food supplies are major vectors of such infections. Appropriate and accurate detection and monitoring technologies are thus of importance in many settings. It is critical in a clinical setting to determine the cause of illness in humans in order to allow appropriate treatment. Detection technologies are also important in the agricultural industry both to ensure food safety and to maximize profitability by avoiding the spread of disease. They are also of importance food and water processing and distribution to ensure safety of food products and water supplies. Finally, pathogen detection is also of importance in warfare and population security given their potential use as biological weapons. Conventional microbiological to detect bacterial toxins [1,2,3,4]. The combination of PCR and ELISA has also been used for detection of pathogens [5,6] and their toxins [7,8]. These methods still require pre-enrichment to increase bacterial concentration above the detection threshold.

A biosensor is an analytical system designed to detect and/or quantify the presence of a specific biological analyte. A biosensor typically integrates a bio-recognition element with a transduction system, as well as electronic systems such as signal amplifiers, processors, and display. Biosensors have been looked upon as alternative for monitoring of bacterial cells and their toxins [9,10]. Platforms such as quartz crystal microbalance (QCM) [11], micromechanical resonators [12,13,14,15,16], flow cytometry [9,17], amperometry [18,19], and surface plasmon resonance (SPR) [20,21,22,23], have been considered. The specificity of biosensors is imparted by a probe such as a nucleic acid, an antibody, an enzyme, a cell or an artificial receptor. Different biological probes such as DNA [24], RNA [25], monoclonal [26,27] polyclonal antibodies [28], bacteriophages [19,29,30,31,32,33] and their recombinant binding proteins [32,34] have been used for detection of bacteria.

Membranes are commonly used in a variety of biomedical applications. They are used as filters for the concentration and isolation of cells, viruses and bacteria, detection of proteins, DNA and RNA in western, Southern and northern blots respectively, and other tests such as direct epifluorescence technique (DEFT). Common materials for membranes include nitrocellulose and polycarbonate. Other materials such as lipid bilayer membranes are also of interest largely for their biomimetic properties. Such membranes are being incorporated into various sensors and biosensors in particular. These biosensors are being used to detect different compounds including proteins, DNA and RNA, bacterial cells and virus particles. Some of these sensors can be and have been used for the detection of pathogens.

This article reviews recent literature specific to the use of membranes for the detection of bacterial pathogens. The review is topically organized inasmuch as first covering the materials science and fabrication techniques employed for the realization of membranes. The next two sections then cover the different molecular probes and the linker chemistries that have been employed in conjunction with such membranes. The last section discusses some of the various platforms that have been used for the readout of these devices.

## 2. Membrane Materials and Fabrication

Membranes are commonly used in biomedical applications. Often they are used either as a filter or as a support structure. Given these applications, there are two general areas of interest for membrane design, physical dimensions and chemical composition. The physical structure generally concerns parameters like surface area, surface roughness, pore size (if any) and distribution and membrane thickness. The physical structure of the membrane is more critical for filtering applications, while the chemical composition is more critical for structural support applications. Given the prevalence of support structure applications, the composition of membranes has been divided into inorganic membranes, organic membranes and more complex hybrid or composite membranes.

### 2.1. Inorganic Membranes

While uncommon, inorganic membranes have been used in numerous biosensor applications. In general the inorganic membranes serve purely as a support structure, but they may also serve to increase the surface area of the sensor or to perform capillary action. In a number of cases inorganic and organic membranes were incorporated together for use in more complex sensing platforms. These will be covered in the hybrid membrane section.

Nanoporous alumina or aluminum anodized oxide membranes have been used in a number of applications [35,36,37,38,39,40,41,42]. In some cases the nanoporous aluminum was only nominally in the form of a membrane since the surfaces were carved into alumina which was deposited on an electrode [38], or sputtered onto the surface and then anodized [40]. Alumina membranes can also be obtained commercially, and one group used commercially available Anodisc inorganic filter membranes from Whatman [41]. Alumina membranes have a number of desirable properties including non-conductivity, well defined nanopores, small pore size, high pore density, and ease of functionalization [39].

Aside from aluminum oxide membranes, gold [43,44], silver [41], titanium oxide [45] and glass [46,47,48,49,50] inorganic membranes have been used. A common application of glass fiber membranes is the transport of fluids by capillary action in more complex membrane based sensors [47,48,49,50]. Silicon nitride has also been used in hybrid membrane structures as a support for organic membranes [51]. In one case a number of different membranes were evaluated. Aluminum oxide, silver and gold-coated polycarbonate track-etched (PCTE) membrane filters were examined to determine the best material and pore size for surface enhanced Raman spectroscopy [41]. Since surface enhanced Raman spectroscopy was used as the transduction method, it is not surprising that the gold membranes performed the best.

### 2.2. Organic Membranes

Organic membranes are more commonly used for biosensor applications than inorganic membranes. The membranes are generally used as a support structure, but they may also be used for filtration or less frequently as an integral component of the sensing process. The first common membrane is nitrocellulose (also known as cellulose nitrate) [47,48,49,50,52,53,54,55,56,57,58,59,60], which is very suitable for many biomedical applications. Other membranes were made with polyethersulfone [54,61,62,63,64,65,66,67,68], polydimethylsiloxane (PDMS) [44], nylon [54,69,70,71,72], polypropylene [73], polylactic acid (PLA) nanofibers [74], cellulose [47,48,49], polycarbonate [75], polyacrylamide [76], cellulose acetate [77,78], polyvinyl chloride [79], polyamine/polyurethane [79] and polyvinylidene fluoride (PVDF) [68].

There are a plethora of commercial organic membranes available and a number have been used. Many of these membranes are sold in a pre-functionalized state. Simple incubation with the biomolecule of interest allows it to be immobilized on the surface of the membrane, either through adsorption or through covalent bonding. Examples include Biodyne B membranes, which consist of nylon functionalized with quaternary ammonium groups, Biodyne C membranes which are also made of nylon but are functionalized with carboxyl groups which render them negatively charged, and Supor PES Membranes, which are made of polyether sulfone and are functionalized to be hydrophilic [68].

One group evaluated nitrocellulose membranes as a visual response membrane sensor involving line formation. Membranes tested include AE 100, AE 98, Immunopore FP, Immunopore RP, HiFlow Plus HF135, HiFlow Plus HFB180, HiFlow Plus 090, and Unisart 140. Interestingly, AE 98 was selected because it provided the best line intensity and shape [55]. Another group compared the results from a number of different membranes including dialysis membranes, which were likely a type of cellulose (material not provided by Fisher Scientific); nitrocellulose, neutral nylon (Biodyne A), positively charged nylon (Biodyne B) from Gibco BRL, and preactivated membranes (ImmunodyneABC and UltraBind) from Pall Specialty Materials. For the detection of *E. coli* subspecies, the best results were obtained using direct protein binding to Immunodyne ABC membranes [80].

A different group compared the performance of Ultrabind membranes to screen-printed carbon electrodes (SPEs) and Maxisorp microtiter wells. The greatest density of bound protein was found on the microtiter plates, while the membranes and electrodes had the highest stability during storage and highest stability during operation, respectively [67].

Many materials and methods were used to manufacture membranes. One interesting example concerns membranes fabricated using polyacrylamide. The polyacrylamide was chosen because of their biocompatibility and hydrophilicity which helps prevent nonspecific adhesion. The monomer concentration was altered to vary the pore size. Glass channels were functionalized with 3-(trimethoxysilyl) propyl acrylate to provide acrylate groups for attachment of the polyacrylamide membranes. The channels were filled with a acrylamide/bisacrylamide/VA-086 photoinitiator solution and a laser was used to form the membrane. The unreacted polyacrylamide was washed through [76].

Common membranes are sometimes modified not for the linking process, but for the transduction process. In one case microporous polycarbonate membrane was modified using polypyrrole modification to create conductive membranes in order to detect Salmonella-infecting phage [79]. In another case cellulose acetate (CA) membranes were grafted with hydroxypropyl cellulose (HPC). The hydroxypropyl cellulose was first crosslinked using divinyl sulfone (DVS) to form branching structures. The cellulose acetate was then reacted with the DVS and then the HPC was grafted onto the CA. The HPC at temperatures below 43 °C expands into a hydrophilic state and above the critical solution temperature of 43 °C collapses into a hydrophobic state. The goal of the HPC (with a low critical solution temperature) is that theoretically, it can be used to decrease fouling of the membranes by using the temperature cycling to “shake off” contaminants [78].

Another method of membrane fabrication is based on nanocomposites. For the purpose of nucleic acid detection, one group fabricated anion exchange nanomembranes that were made up of quaternary ammonium containing divynylbenzene/polystyrene particles embedded in a polyethylene-polyamide/polyester matrix for mechanical stability [81]. In a different set of experiemnts, nitrocellulose particles were embedded in a cellulose acetate matrix. The nitrocellulose viscosity and concentration, and the cellulose acetate concentration were varied to alter the capillary flow rate and maximize protein binding [56].

Membranes were also formed using nonwoven fibers. In one case nonwoven polypropylene microfibers were obtained and polymerized with pyrrole and 3-thiopheneacetic acid using FeCl_3_ and doped with 5-sulfosalicylic acid [73]. Another group used electrospinning to produce nanofiber nitrocellulose membranes. Parallel electrodes were used to create aligned mats of nanofibers to enhance capillary action [59,60].

Many applications are based on the use of lipid bilayer membranes, often to better emulate or make use of physiological conditions. Some applications made use of membrane engineering [82,83,84] of live cells in order to use them for biosensor applications, while others created biomimetic lipid bilayer membranes [51,85,86,87,88,89] to emulate the physiological conditions. One method for membrane engineering is through electroinsertion of antibodies to embed the desired antibodies into the cell membrane [83,84].

In another case, planar tethered bilayer lipid membranes were used for bacteria detection. The lipid membranes were anchored to the gold surface using a gold-sulphur bond and the silane surface through the hydrogen bonds of a silane-hydroxyl bond. 2,3-di-O-phytanylglycerol-1-tetraethylene glycol-D,L-lipoic acid ester lipid, 2,3-di-Ophytanyl-sn-glycerol-1-tetra-ethylene glycol-(3-tryethoxysilane) ether lipid, and cholesterolpentaethyleneglycol were used for self-assembly of the first half of the membranes, while the second half was deposited using vesicles composed of 1,2-di-O-phytanoyl-sn-glycero-3 phosphocholine and cholesterol. Such assemblies allowed the specific detection of toxins associated to pathogenic bacteria [51].

In a different case, liposomes were used directly for the detection of cholera toxin and to transduce it into a visible output. The liposomes were formed by combining ganglioside GM1 and 5,7-docosadiynoic acid with a solvent, sonicating the solution, and causing polymerization to take place using UV radiation. Introduction of cholera toxin into the liposomes leads to a change in their light absorption [88].

Another group created a biomimetic membrane from tryptophan-modified 10,12-tricosadiynoic acid (TRCDA) and 1,2-sn-glycero-dimyristoyl-3-phosphocholine (DMPC) in agar and liquid media. The TRCDA creates polymers when exposed to UV light. It also creates a colourimetric change when TRCDA polymers are exposed to mechanical stress, changes in pH, binding of biological agents or heat. TRCDAs have been used in vesicles for detection of nucleic acids, proteins and microorganisms [89].

### 2.3. Hybrid Membranes

While many membranes are clearly composed of organic or inorganic components, some hybrid membranes have inorganic and organic materials which are effectively fused together. One example is gold-coated polycarbonate track etched (PCTE) membrane filter which was used for Surface Enhanced Raman Spectrometry-based detection of Giardia [41].

One simple example of the hybrid membranes was a PDMS membrane coated with 20 nm gold to allow linking of thiols to the surface [44]. A different group also used gold, but the membranes where formed on the inorganic surface in this case. Liposomes were formed using 1,2-dipalmitoyl-sn-glycero-3-phosphocholine (DPPC), which is commonly found in cells and is therefore useful for a biomimetic application, to create a phospholipid membrane on the gold electrode surface. The liposomes were simply introduced in solution to the gold surface to form the membranes [90]. DPPC liposomes containing monosialoganglioside (GM1) have also been deposited on octanethiol attached to gold to form a GM1 containing phospholipid bilayer [86]. A similar method has been used except that the gold electrode was prepared using thiol-containing molecules octanethiol, 1,2-dimyristoyl-sn-glycero-3-phosphothioethanol or spacerlipid A (created by the authors), after which the phospholipid was deposited through vesicle fusion. Such assemblies were employed to detect the presence of Clavibacter through the monitoring of related cytotoxins [85].

Membrane formation can also be achieved through sol-gel methods. This was used to make nano-TiO_2_ and nano-TiO_2_-polyethylene glycol membranes. A solution of Ti(OBu)_4_ (with polyethylene glycol for the second membrane) in acetic acid was added to a solution of condensed HCl, water, DMF and alcohol and allowed to condense. The resulting gel was placed on the electrode by dip-coating [45].

Another interesting method involves the formation of a bilayer lipid membrane through the activation of an egg phosphatidylcholine, hexadecylamine and cholesterol solution by KCl on top of an agar-coated Teflon surface [87].

### 2.4. Composite Membranes

**Figure 1:**
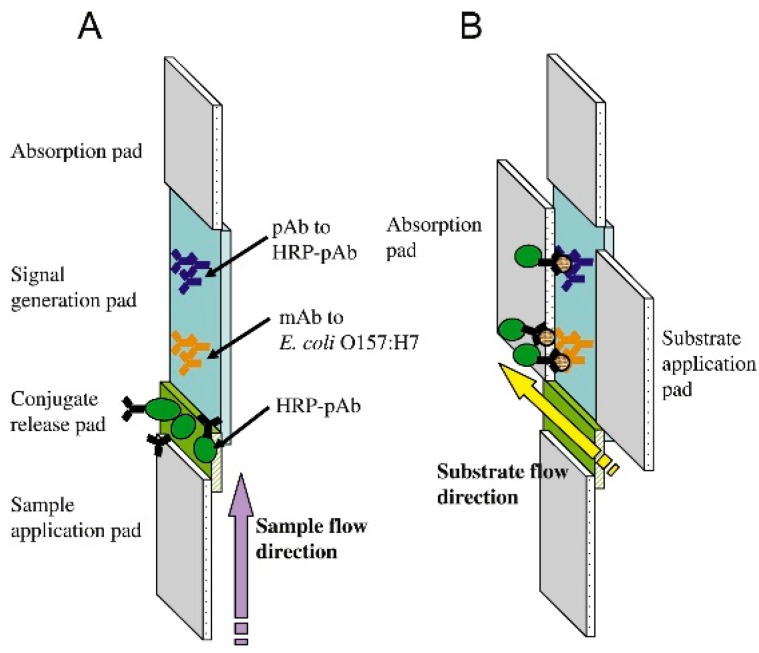
A composite membrane sensor. (**A**) The liquid sample containing the *E. coli* is placed on the glass fiber membrane sample application pad. The solution flows towards the cellulose membrane absorption pad. Along its path HRP conjugated polyclonal antibody (HRP-pAb) enters the solution as it is released from the glass fiber conjugate release pad. Some of the HRP-pAb binds to the *E. coli*. The pathogen with attached HRP then binds to the monoclonal antibody (mAb) bound to the nitrocellulose membrane signal generation pad. Some unbound HRP-pAb binds to the pAb to HRP-pAb as a control; (**B**) A reaction then takes place with a substrate solution which is catalyzed by the HRP to produce a visible output. With permission from [47].

Composite membranes consist of multiple different membranes which are sandwiched together vertically or side to side to form a complete sensor. One group used a sample application pad consisting of a glass fibre membrane, a conjugate release pad made of a glass membrane, a signal generation pad made of a nitrocellulose membrane, and an absorption pad made of a cellulose membrane. Such devices were employed to monitor the presence of bacterial pathogens such as *E. coli* 0157 and *Yersinia pestis* [47,49,50] (Figure 1).

**Figure 2:**
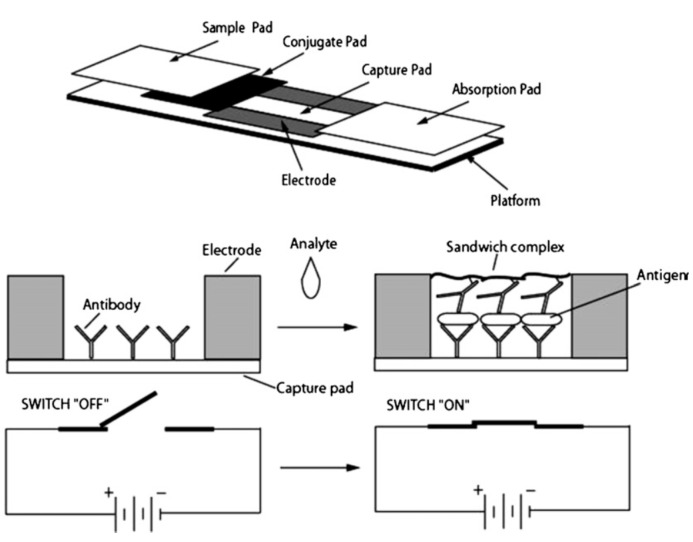
A composite membrane biosensor with a membrane composition similar to that in Figure 1. The pathogen was introduced in solution to the cellulose membrane sample pad. This solution flowed towards the absorption pad, also made of cellulose. Along the way the conductive material-conjugated antibodies were released from the fiberglass conjugate pad and bound to the pathogen. These pathogens then bound to the antibodies linked to the nitrocellulose capture pad and increased the conductivity of the circuit. With permission from [91].

A similar design consisted of sample and absorption pads made of cellulose membranes, a fiberglass membrane for the conjugate pad and a nitrocellulose membrane for the capture pad [48,91]. Instead of the visual output however, electrodes were also included beside the capture pad. Then polyaniline [91] (Figure 2) or iron oxide nanoparticle [48]-conjugated antibodies were used to detect the antigen, and form an electrical circuit.

## 3. Molecular Probes

In order to specifically detect the pathogens of interest, it is generally necessary to use a sensing molecule or molecules which bind only the pathogen or component of the pathogen of interest. Like many biosensors, antibodies [37,39,41,42,47,48,49,50,53,54,57,59,60,68,70,71,73,75,76,77,78,82,83,84,91] are the most common reagent for specific detection of biomolecules. Often two antibodies may be used, one to specifically capture the pathogen and a second conjugated antibody which can be used to provide transduction into an observable output. In some cases, such as when piezoelectric sensing is used, the secondary antibody can also be used to amplify the signal output [37]. The use of specific DNA or RNA probes for oligonucleotide hybridization with extracted DNA or RNA from the pathogen of interest has also been reported [35,36,38,40,43,45,46,55,58,61,62,63,64,65,66,73,81,87].

**Figure 3:**
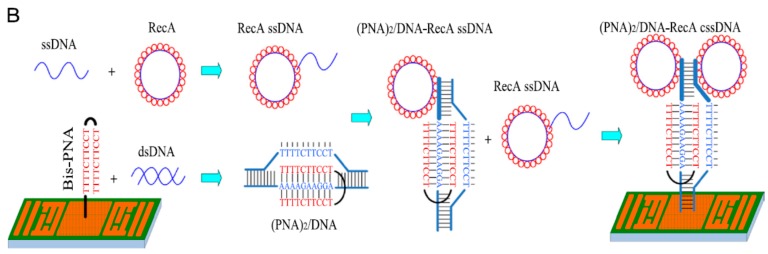
A complex DNA hybridization scheme. A bis-PNA DNA structure was used to specifically detect dsDNA from a pathogen. The mass change from this interaction is small however. In order to improve the detector sensitivity, single stranded DNA (ssDNA) linked to protein RecA was used to amplify the mass change while maintaining specificity as the ssDNA hybridizes only with the complex DNA structure already formed on the sensor surface. With permission from [43].

Some of the nucleic acid hybridization schemes were more complex than others, however. In one case a more complex DNA structure called a bis-peptide nucleic acid (PNA) was used, which involved a looped complementary DNA structure. This structure undergoes hybridization with double-stranded DNA (dsDNA) from the pathogen, and a single stranded DNA probe linked to a RecA protein which is used to increase the biosensor sensitivity [43] (Figure 3). Another interesting method to increase sensitivity was through the use of short sensing DNA probes which were used to detect longer strands of pathogen DNA. The sensitivity was then increased by using PCR to extend the probe DNA to the length of the pathogen DNA [36].

There are also specific protein interactions which may be used for pathogen detection. Cholera toxin, for example, binds to ganglioside GM1, and this interaction can be used to determine the presence of cholera [86,88]. Another method of specific detection is through the use of viruses called bacteriophages which may be used to detect specific bacteria [79].

A number of other more unusual, and generally less specific, methods have also been employed for pathogen sensing applications. One group used a phospholipid membrane to detect spontaneously inserting protein channels from *Clavibacter michiganense ssp. Nebraskense* [85]. Two papers describe the use of arrays of lectins to identify pathogens. Lectins are proteins which bind to carbohydrates. In the first paper, ten different lectins were immobilized onto membranes. A solution containing one of four different *E. coli* strains was introduced to each different lectin coated membrane, making 40 combinations in all. A solution containing ferricyanide, succinate, formate and menadione in growth medium lacking proteins and trace elements was added to each membrane. After incubation the ferrocyanide was detected by chronocoulometry. The change in charge for each of the 40 combinations along with statistical analysis was used to differentiate each of the four different *E. coli* strains. In fact, the authors found that only five lectins were necessary to distinguish between the four subspecies [72]. In a different paper, the same method was used to distinguish between *E. coli*, *S. aureus*, *S. cerevisiae*, *B. cereus*, *P. vulgaris*, and *E. aerogenes*. In addition to 10 lectins, BSA and control membrane binding was also tested. As before, chronocoulometry was used in addition to statistical analysis to differentiate between the different bacterial species [80].

There have been some reports of less specific and non-specific detection methods which may nevertheless provide useful information. In one case, 11-mercapto-1-undecanoic acid (MUA), 11-mercapto-1-undecanol (MUO) and dodecane thiol (DOT) were investigated for *E. coli* detection. The authors were able to determine if the *E. coli* cells were alive or dead [44]. In another case, the authors were able to distinguish between catalase-positive and catalase-negative bacteria by the detection of hydrogen peroxide production [69]. Thirdly, the lectin concanavalin A was used to detect dengue glycoproteins [90]. Lipids have also been used for the detection of bacterial toxins through pore formation in the lipid bilayer [51]. Tryptophan-derivitized TRCDA was used for the detection of several species of bacterial cells [89]. One group used protein phosphatases to detect microcystins which are produced by cyanobacteria [67].

## 4. Linking Procedure

Another important aspect of biosensors is the method that is used to link the capture molecule to the surface, in this case a membrane. This is important because it can substantially affect the sensitivity and specificity of the biosensor. Glutaraldehyde is a simple short crosslinking molecule, and it is commonly used to link two amine groups together. Often one of these is a free amine group on an antibody. It was used to link antibodies to secondary amines in a polypyrrole containing polymer membrane [73,80], a nitrocellulose membrane [59,60,91], Biodyne B membranes [71] and polycarbonate membranes which were aminated using nitric acid and sodium borohydride [75]. Glutaraldehyde was also used to attach aminated DNA probes to an aluminum oxide surface aminated by 3-aminopropyltrimethoxysilane [36,38,40].

One group compared three linking procedures to attach protein phosphatase (PP) to Screen-printed carbon electrodes (SPEs). The first method, was performed using a sol gel formed by tetramethoxysilane (TMOS), methyltrimethoxysilane (MTMOS), polyethylene glycol (PEG600) and hydrocholic acid. This was mixed with the PP and deposited on the electrode. In the second method, glutaraldehyde was added to a solution of BSA and PP on the surface of the electrode. For the third method, photocrosslinkable poly (vinyl alcohol) bearing styrylpyridinium groups (PVA-SbQ) were mixed with the PP and applied to the electrode. As it resulted in the highest density of the PP enzyme on the surface, this method was also used on two other surfaces, Maxisorp microtiter wells and Ultrabind polyethersulfone affinity membranes [67].

Streptavidin and biotin is a pair of very tightly binding molecules which are commonly used in linking procedures [92]. Streptavidin is usually adsorbed directly to a surface, while the molecule of interest is linked to the biotin. For these sensors strepavidin was used exclusively for linking DNA or RNA to membrane surfaces, but it may be used for antibody linking as well. For instance, biotinylated DNA [61,62,63,64,65] or RNA [66] was linked to streptavidin adsorbed on the membrane surface.

Silanes constitute another group of molecules which are very commonly used in linking processes using a variety of surfaces. Several of the previous linking processes included a silane as a component of the functionalization process. DNA [35] or antibodies [37,39] have been linked to an alumina surface using (3-glycidoxypropyl) trimethoxysilane. Another group incorporated a silane group into their lipid (2,3-di-ophytanyl-sn-glycerol-1-tetraethylene glycol-(3-triethoxysilane)) to anchor a biomimetic membrane to a silicon nitride surface [51].

Other work with lipid bilayers involved living cells which were functionalized with antibodies. This was performed using electroporation, which through the application of an electric potential to the cells causes pores to form in the cellular membrane. Antibodies can then attach themselves in these pores [82,83,84].

Another very popular chemistry for crosslinking involves 1-ethyl-3-(3-dimethylaminopropyl) carbodiimide (EDC) and N-hydroxysulfosuccinimide (NHS) or sulfo-NHS. EDC binds free carboxyl groups and amino groups. Sulfo-NHS enhances the effectiveness of EDC and binds to EDC after it binds a carboxyl group and a free amino group. Hyaluronic acid was used to modify an alumina surface and create free carboxyl groups. Then EDC/sulfo-NHS chemistry was used to immobilize antibodies [42]. Carboxyl groups were formed on the quaternary ammonium containing divynylbenzene/polystyrene particles embedded in a polyethylene-polyamide/polyester matrix by reaction with benzophenone tetracarboxylic acid and exposing it to UV light. EDC was then used to link the carboxyl groups to aminated oligonucleotide probes [81]. A nylon membrane was first incubated sequentially with dimethyl sulfate and 6-aminocaproic acid solutions to provide free carboxyl groups on the surface. EDC/sulfo-NHS chemistry was then used to link the antibodies to the nylon surface [70].

EDC can also be used without the addition of sulfo-NHS, for example to link antibodies to a hydroxypropyl cellulose membrane [78]. Linking can also be performed with other carbodiimides. One group linked antibodies to Biodyne C membranes using 2-ethyl-5-phenylisoxazolium-3′-sulfonate (Woodward’s reagent) [71].

One of the simplest linking methods is based on sulfide groups, which link naturally to gold surfaces. Sulfide groups were introduced to DNA strands [43], small organic molecules [44], and lipids to link them to gold surfaces [51]. Hydrophobicity can play a major role in the linking process. Protein can adhere to a phospholipid membrane simply through incubation [90]. Liposomes will naturally bind to a surface which is modified to be hydrophobic [86], and GM1 ganglioside will naturally be incorporated into liposomes during formation [88]. The addition of a hydrophobic dodecane tail to a DNA probe can be used to anchor it into a bilayer lipid membrane [87].

In some cases, complex linking process are unnecessary and simple adsorption of antigen or antibody to a nitrocellose [47,48,49,50,53,54,55], glass fibre [47,48,50,91] or cellulose [47] membrane is sufficient The same concept applies to adsorption of DNA to a sol-gel deposited membrane [45] and horseradish-peroxidase conjugated antibodies to a nitrocellulose membrane [57]. This may even apply to certain components of more complex linking processes.

**Figure 4:**
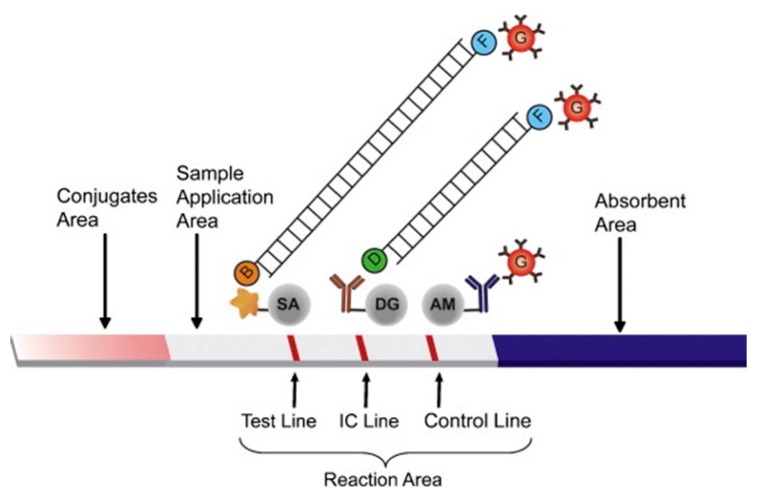
An example of a more complex linking process. Microspheres coated with streptavidin were adsorbed onto glass membranes. Forward primers were labeled with biotin while reverse primers were labeled with fluorescein. Upon pathogenic DNA binding and annealing, the ds-DNA labeled on one 5′ end with biotin which binds to the microspheres on the surface, and the other 5′ end with fluorescein to which gold nanoparticles coated which antibodies specific to fluorescein can bind. These gold nanoparticles give a visual colour change which can be observed with the naked eye. The other two lines serve as controls to ensure proper conditions for PCR amplification (internal amplification control) and release of the antibody coated gold particles. The abbreviations are: streptavidin-coated microspheres (SA), biotin (B), fluorescein (F), antidigoxigenin antibody-coated microspheres (DG), digoxigenin (D), goat anti-mouse IgG antibody-coated microspheres (AM), and anti-fluorescein antibody-conjugated gold nanoparticles (G). With permission from [46].

**Figure 5:**
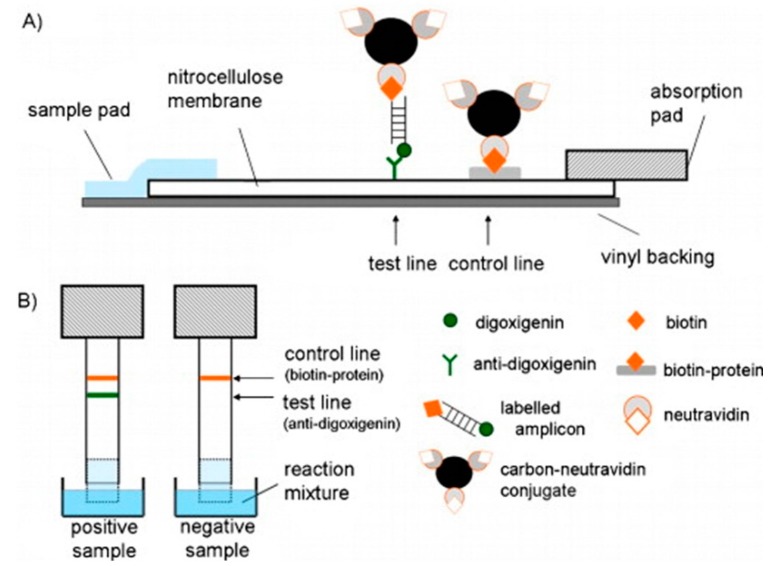
Similar membrane sensor to that shown in Figure 4. Labeled PCR amplicons are linked to the membrane surface through anti-digoxigenin antibodies. Neutravidin coated carbon particles link to the other end of the labeled amplicons, which is visible to the naked eye. The anti-digoxigenin and biotin-protein complex were adsorbed to the nitrocellulose membrane. With permission from [55].

PCR is commonly used to amplify DNA or RNA from pathogens for detection, and was also employed in a number of the biosensors discussed here. In one case, it was employed in an intriguing manner for specific detection of DNA. Microspheres coated with streptavidin were adsorbed onto glass membranes. Forward primers were labeled with biotin while reverse primers were labeled with fluorescein. If DNA is present, then after annealing, the ds-DNA would be labeled on one 5′ end with biotin which binds to the microspheres on the surface, and the other 5′ end with fluorescein to which gold nanoparticles coated which antibodies specific to fluorescein can bind. These gold nanoparticles give a visual colour change which can be observed with the naked eye [46]. (Figure 4) Similar work was performed by replacing the fluorescein with digoxygenin which is bound by antibody to the surface and replacing the gold nanoparticles with carbon nanoparticles. The anti-digoxigenin and biotin-protein complex were adsorbed to the nitrocellulose membrane (Figure 5) [55].

As discussed in Section 3, there are a number of prefunctionalized membranes which are available commercially. These include membranes like Immunodyne ABC and UltraBind membranes, which respectively feature nucleophile-selective and aldehyde-activated surfaces [80]. The Immunodyne ABC membranes for example were used to directly form covalent bonds with free amine groups on proteins introduced to the membranes, though the precise reaction was unspecified by the manufacturer [72].

Frequently membranes are not used purely as a support, but also as a filter [41,58,68,76,79]. Simple adsorption or collection of viruses and bacteria may be sufficient for the biosensors so that linking to the surface is unnecessary. The membranes were used for filtration in order to trap the cells while allowing unbound nanoparticles to pass through the pores in the membranes [77].

For some applications the membranes may even be used in reverse. One group, for example, used a nylon membrane in a catalase activity sensor to prevent biofouling by bacterial buildup on the sensor [69].

## 5. Transduction Systems

The transduction process is the method by which the biochemical interaction of the capture molecule and the target pathogen is converted into an observable output signal. Ideally this output can also be used to determine the concentration of the pathogen being detected. Numerous different detection methods have been employed. The most popular by far were those involving electrical or optical phenomena.

### 5.1. Electrical

Many transduction methods involve observation of a change in the electrical conditions in the system. Frequently, the simple act of binding of detection molecule and antigen can lead to a change in the electrical characteristics of the biosensor, including the potential, current, resistance, and/or impedance. Oligonucleotide hybridization on a positively charge nanomembrane [81] and glycoprotein binding to a membrane [90] were observed and quantified using electrodes and the resultant current vs. voltage (resistance) plot. Another group quantified bacterial binding to a membrane by recording the change in membrane resistance upon binding [73].

Impedance can likewise be used to record antibody-pathogen binding on a membrane.[37] In another case impedance spectroscopy was used to characterize the formation of spontaneously inserting anion channels from bacteria in biomimetic membranes [85].

A change in electrical current is often observed in transduction methods. Catalase-positive and -negative bacteria were identified by hydrogen peroxide consumption. The hydrogen peroxide was detected amperometrically using a graphite–Teflon–peroxidase–ferrocene electrode [69]. In another study, a change in current of the modified patch-clamp pipette electrode was recorded, which allows changes in concentration of nucleic acids to be determined [87]. Bacteria bound to membranes were detected electrochemically by cellular uptake of ferrocyanide through chronocoulometry [80]. Another group used antibodies to bind *E. coli* cells to a membrane. Horseradise peroxidase-conjugated antibodies were then used to detect the *E. coli* cells as in a sandwich ELISA (enzyme linked immunosorbent assay). NaI, ortho-phenylenediamine and hydrogen peroxidase substrates caused a current to develop which allowed for quantification of the *E. coli* cells [71].

Like the other electrical characteristics, potentiometric-based transduction methods are also common. The attachment of viruses to antibodies in a cellular membrane leads to a change in potential that can be recorded via electrodes [83,84].

One novel transduction method makes use of live cells which have been engineered to detect specific pathogens. Virus particles bind to specific antibodies which have been inserted into the cellular membrane. This results in a change in membrane potential which can be detected using an electrode and a voltmeter [82].

Similarly, several methods were tested for detection of bacterial toxins through lipid damaging or pore formation effects in lipid bilayers. These effects change the ion permeability of the membranes which can be detected by electrochemical impedance spectroscopy or plasmon resonance spectroscopy. *Staphylococcus aureus* (MSSA476), *Pseudomonas aeruginosa* (PAO1) and *E. coli* (DH5α) showed ion permeation through change in impedance [51].

**Figure 6:**
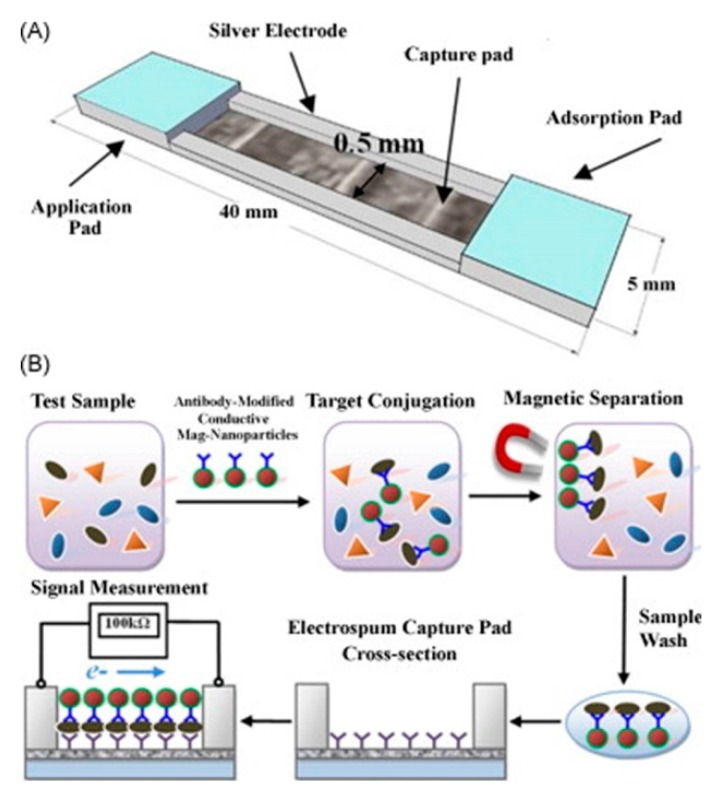
(**A**) Schematic of the biosensor structure and membrane assembly consistingof cellulose application and absorption pads and electrospun cellulose nitratecapture pad; (**B**) Detection scheme of the lateral flow immunosensor based on theantibody-functionalized electrospun capture membrane. With permission from [59].

Another more unusual transduction method is based on the use of antibodies conjugated to conductive materials to close an electrical circuit. In this approach, pathogens are first immobilized on membranes. The device is then exposed to conductive polyaniline- [91] or iron oxide nanoparticle- [48] conjugated antibodies which will specifically bind to the target and close the electrical circuit. The change in resistance and conductance, respectively, were used to quantify the pathogens. In different papers, polyaniline and iron oxide nanoparticle -conjugated antibodies were used to concentrate the bacteria and viruses using magnetic separation. The antibody covered bacteria and viruses were then bound to nitrocellulose nanofilament membranes using secondary antibodies, and the change in resistivity was used to determine the concentration of bacteria or viruses [59,60]. This sensor’s operation is shown in Figure 6.

Often membranes may serve as a physical support in the biosensors. Conversely, the properties of porous membranes may be harnessed directly by various electrical transduction methods. DNA hybridization in the membrane pores leads to blockages which can be detected with electrochemical impedance spectroscopy (EIS) [35]. Sensitivity can be increased by linking gold nanoparticles to the pathogen DNA and by silver catalytic deposition. In a different experiment, DNA hybridization in alumina nanochannels was shown to restrict the binding of ferrocyanide to an electrode. This results in a decrease in peak current in cyclical voltammograms which can be used to quantify the DNA [38]. In another similar experiment, DNA hybridization was again used to create ion blockages in alumina membranes. As before, the hybridization of the probe and target DNA took place in the pores of the membranes, and cyclic voltammetry and EIS were used to quantify the DNA. In addition to the hybridization process, however, Taq DNA polymerase was used to elongate the 20 base pair probe sequences to the length of the full target DNA sequence. This led to a substantial enhancement of the ion blockage, thus increasing the sensitivity of the sensor [36].

Similar work was performed using viral RNA, porous alumina, differential pulse volatammetery (DPV) and ferrocyanide. The transduction process works such that the DPV oxidative peak current decreases as the virus RNA concentration increases [40]. In another study, antibody-pathogen binding in pores led to changes in impedance which were analyzed by normalized impedance change [42]. Bacterial cells have also been detected in a similar manner. The bacteria were captured using antibodies attached to the membranes. This blocked the pores in the membrane, and thus the flow of the electrolyte, leading to an increase in the impedance of the sensor. The impedance was monitored by an impedance analyzer [39].

In a different, more direct, application of membranes, conductive membranes were used as a filter to capture *Salmonella* bacteria. Subsequently, the change in conductivity was recorded to quantify the *Salmonella* bacteria [79].

### 5.2. Optical

The other common basis for many transduction methods is optical phenomena. These types of transduction processes may be characterized by a colour change which is visible to the naked eye (and may be quantified by a reflectometer or microplate reader), photoluminescence (including fluorescence), chemiluminescence, absorbance, or radioactivity. It also includes the use of external methods such as surface enhanced Raman spectroscopy, and interferometry. Although these may also include some kind of electrical transduction method, such as a CMOS image sensor, they have been classified as optical as the primary output of the sensors is optical in nature.

#### 5.2.1. Color Change

A visual colour change is one of the major optical transduction methods. In some cases it was used as a method of detection by the naked eye, while in others a reflectometer or absorbance measurement was performed for quantification. A number of experiments have been performed with liposome-conjugated DNA probes which were used to detect pathogen DNA/RNA. Short single stranded DNA or RNA probes were used to link single stranded DNA or RNA from the pathogen to a membrane. Subsequently, DNA-linked liposomes containing a dye were used to label the other end of the pathogen’s DNA/RNA. The dye was then released, and the colour change was quantified via a reflectometer [61,62,63,64,65,66].

Another way to produce a visual colour change is through the attachment of gold [46] or carbon [55] nanoparticles to sensing molecules (Figure 3 and Figure 4). Similar work was performed using commercially available carboxylated magnetic nanoparticles. These particles were coated with BSA using EDC/NHS chemistry. Through electrostatic attraction, gold nanoparticles were attached to the BSA on the magnetic nanoparticles. Subsequently, antibodies were attached to the composite nanoparticles through physical adsorption. These nanoparticles were used to bind to bacteria, and filtered through the membrane. The bacteria with bound particles were trapped while the unbound nanoparticles passed through the pores of the membranes. The bacteria were then detected by visual colour change caused by the aggregated gold nanoparticles, and this colour change was enhanced by the addition of hydroxylamine and HAuCl_4_ [77].

Horseradish peroxidase (HRP) is commonly used to catalyze colourimetric reactions. HRP-conjugated antibodies bind to the pathogen, and the addition of substrates such as tetramethylbenzidene [47,75] and SuperSignal West Femto [47] in addition to hydrogen peroxide allow the colourimetric reaction to take place. Protein phosphatases (PP) may also be used to catalyze colourimetric reactions, as seen in an experiment with Microcystins. Microcystins are toxic proteins produced by cyanobacteria such as *Microcystis*, *Anabaena*, *Oscillatoria* and *Nostoc* which inhibit the activity of protein phosphatases. In this experiment, PP were immobilized on the membrane surface, and microcysteins were introduced in solution. Subsequently, colourless *p*-nitrophenyl phosphate (*p*NPP) was introduced in the PP. This converts the pNPP to yellow *p*-nitrophenol (*p*NP), which can be used to measure enzyme activity using a microplate reader, which in turn can be used to determine the presence of microcystins [67].

In a different experiment, lectin proteins on a membrane were used to bind bacterial cells. Subsequently, the oxidants menadione and ferranocyanide, and the respiratory substrates formate and succinate were introduced to the cells. Oxidation of the ferranocyanide led to a change in colour which was related to the quantity of bacteria [72].

Two of the most unusual transduction methods involved colour changes based directly on interaction with the molecule of interest. In the first, polydiacetylene liposomes with embedded ganglioside GM1 were used to detect cholera toxin. The cholera toxin binds to GM1, and the binding interaction causes conformational changes in the polymer backbone of the liposomes. This results in a change of the liposome colour [88]. Similarly, tricosadiynoic acid (TRCDA) vesicles change colour when exposed to lipolysaccharides from pathogens and other microorganisms [89].

#### 5.2.2. Light Emission

**Figure 7:**
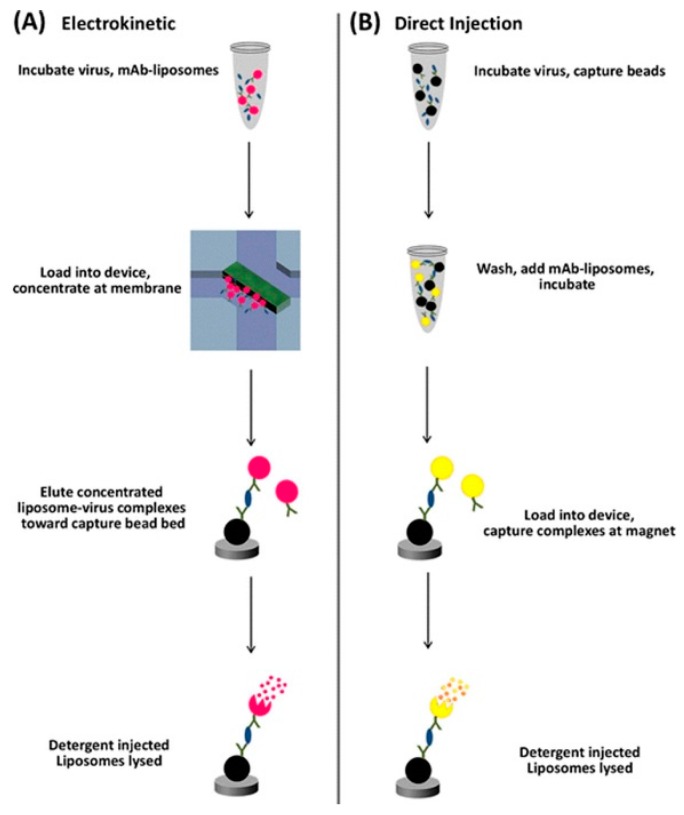
Detection of virus particles with (**A**) and without (**B**) a concentration step using a membrane. The concentration step led to an order of magnitude better sensitivity. With permission from [76].

The emission of light is the other main transduction method used with membrane based pathogen detection. As with colour change, liposomes filled with fluorescent dye may be used for transduction. In this case fluorescent dye-filled liposomes attached to antibodies were mixed with antigen and electrokinetically concentrated using a voltage applied across the membrane before being introduced to the capture bed. Detergent was used to release the fluorescent dye for quantification through video recording (Figure 7) [76].

Other particles may also be linked to antibodies for transduction. CdSe/ZnS core/shell dendron nanocrystals were functionalized with antibodies and bound to antigen on the membrane surface creating a “sandwich”. The crystals are photoluminescent, which allows for quantification of the antigen [70]. In a similar case, the secondary antibodies were conjugated to up-converting phosphor particles, a 980 nm laser was used to excite the phosphor particles and the resulting 541 nm wavelength luminescence was detected using a photomultiplier tube [50].

**Figure 8:**
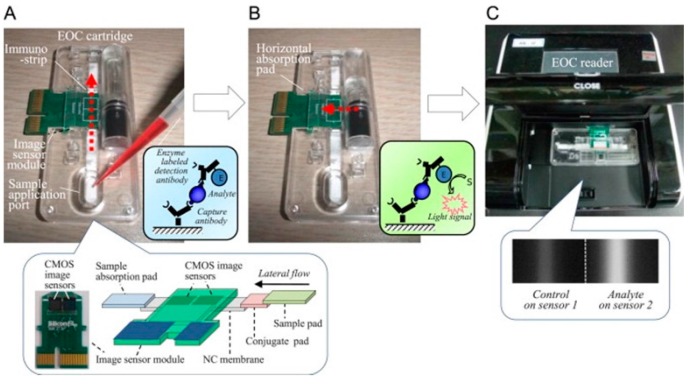
ELISA on a chip reaction with chemiluminescent output and lens free CMOS sensor. (**A**) The substrate is added to the sample pad, the HRP-conjugated antibody is released from the conjugate pad and it subsequently binds to the antigen. The antibody-antigen complex then binds to the capture antibody on the nitrocellulose membrane; (**B**) The luminol and hydrogen peroxide substrates are injected into the reaction chamber and the reaction is catalyzed by the HRP enzyme; (**C**) The chemiluminescent output is recorded by the CMOS sensor and used to quantify the antigen concentration. With permission from [49].

In addition to catalyzing colourimetric reactions, HRP can also be used to catalyze light emitting reactions. In one case, HRP-conjugated antibodies were used to detect virus particles and a luminol-based chemiluminescent reaction used to optically quantify the virus concentration [54]. In a different experiment superparamagnetic beads were linked to antibodies and magnetically attached to a capture bed. Subsequently, bacterial cells were labeled with peroxidase-conjugated antibodies and introduced the antibodies on the capture bed. Once again a catalyzed luminol and peroxidase-based reaction led to a chemiluminescent output which was recorded using a luminometer [68]. In a similar process, also based on a sandwich configuration, a HRP-conjugated antibody and luminol, recorded the chemiluminescence using a lens free CMOS image sensor (Figure 8) [49].

Fluorescently-labeled antibodies may also be used for emission-based transduction. In this case bacteria were introduced to antibodies bound to a membrane and Alexa fluor-conjugated (H + L) antibody fragments were used to detect the bacteria attached to the membrane. This output was observed by a fluorescence microscope [78].

A somewhat more antiquated transduction method (due to safety issues rather than accuracy) is through radiolabeling. Bacterial cells were captured by filtering through a nitrocellulose membrane, and the cells were chemically lysed. Radiolabeled DNA probes were added to bind to the DNA from the lysed cells. The autoradiography was recorded using autoradiography film [58].

#### 5.2.3. Spectroscopic/Interferometric

Other than the inherent characteristics of the system, external light sources may also be used in transduction methods. In the first case, unusually, the mechanical deflection of the membrane was observed. A white light interferometer and a fiber optic interferometer where used to determine deflection of the membranes caused by the binding of the pathogen [44]. In the second case, membrane filters were used to capture bacteria and antibody-coated gold nanoparticles were used to label the bacteria, and were detected using surface enhanced Raman spectroscopy [41].

### 5.3. Other

Aside from electrical and optical transduction systems, several other methods have also been used. A commercial leaky surface acoustic wave system was used to detect pathogen DNA [43] Surface acoustic wave biosensors generally emit a wave through a material via the piezoelectric effect. Binding of biomolecules to the surface of the sensor increases the mass of the material, which leads to a change in the velocity of the wave. This change in velocity can be observed in a number of different ways. One such method is to record the change in time it takes the wave to progress through the material.

Piezoelectric quartz crystals are also popular for mass sensing. The binding of molecules to the surface changes the resonant frequencies of the quartz crystal. This change in frequency can be used to determine the quantity of bound antigen. In this case quartz crystals were used determine the quantity of bound bacterial DNA and cholera toxin [45,86].

**Figure 9:**
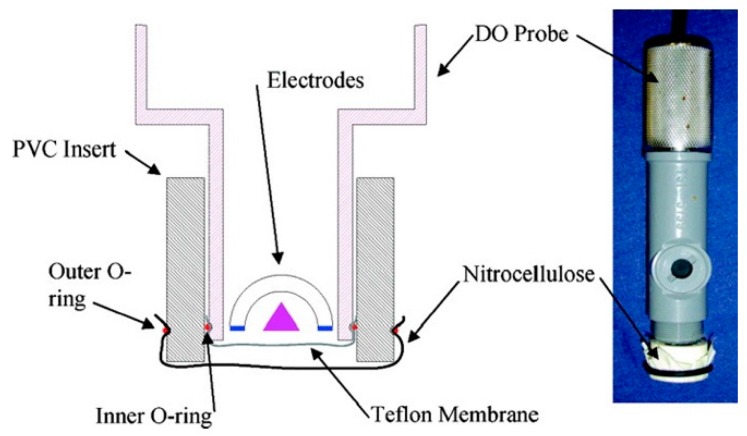
Oxygen sensor probe. With permission from [57].

Magnetic beads coated with antibodies and used to simultaneously detect two virus strains. The beads were measured using a magnetic reader using two frequency magnetic excitation [53]. An oxygen meter was used to detect *E. coli* cells. HRP-conjugated antibodies specific to *E. coli* cells were immobilized onto nitrocellulose membranes. These membranes were fixed above Teflon membrane of the oxygen meter. Hydrogen peroxide was used to produce the oxygen, which was then recorded by the oxygen meter. The production of oxygen was amplified using 2,2′-azino-bis(3-ethylbenzothiazoline-6-sulfonic acid) diammonium salt. When the *E. coli* cells were added to the solution, they reduced access of the HRP to hydrogen peroxide, decreasing the production of oxygen [57] (Figure 9).

## 6. Detection Thresholds

A wide array of different pathogens have been detected, largely through DNA, RNA or whole bacteria or virus particles. It can be difficult to directly compare the different detection limits because the units are different. It is important to note that the whole bacteria and virus particles are much more massive than their DNA or RNA, which means that the DNA and RNA concentrations in a sample will naturally be much smaller in mass/volume, unless they have been amplified (usually through PCR). This is important because in direct mass/volume comparisons, the DNA/RNA methods will almost inevitably appear to be more sensitive for this reason, even though the mass of the virus, and especially the bacteria, in a sample will be much greater than that of the DNA/RNA produced from those viruses or bacteria in a DNA/RNA sensor. Table 1 summarizes the various approaches reported in the literature employed along with the cited detection limits.

**Table 1:** A summary of the pathogens detected, form of detection, detection limit or range where given. Unit abbreviations are colony forming units (CFU), cell culture infective dose (CCID) and plaque forming units (PFU).

Pathogen	Detection Type	Membrane Sensor	Transduction Method	Detection Limit or Range
*Bacillus Anthracis*	RNA	Polyethersulfone membrane with linked ssDNA probe	Reflectometer-based detection of dye-filled liposome linked to reporter DNA probe	1 nM [62]
	RNA	Polyethersulfone membrane with linked ssDNA probe	Reflectometer-based detection of dye-filled lyposome linked to reporter probe	1.5 fmol [64]
*Baccilus Cereus*	whole bacteria	Immunodyne ABC membranes with various linked lectins	Chemometric data analysis of pathogen binding chronocoulometry results were used to distinguish between different pathogens	not given [80]
	whole bacteria	Composite sensor composed of glass fiber, cellulose and nitrocellulose membranes with linked capture antibodies	Detection of antigen with conductive polyaniline nanowire-conjugated antibodies and quantification via change in conductance	10 CFU/mL [91]
Bovine viral diarrhea virus	virus particle	Nanofiber nitrocellulose membranes with linked antibodies	Pathogens coated by conductive nanoparticle-conjugated antibodies were immobilized on the membrane and quantified via the change in resistance.	10^3^ CCID/mL [59]
*Brucella*	RNA	Nanomembrane composed of polystyrene-divinylbenzene particles with quaternary ammonium groups and polyamide/polyestertextile fiber embedded in polyethylene with linked oligonucleotide probe	Change in ion current with oligonucleotide hybridization	1 pM [81]
Cherry leaf roll virus	virus particle	Cellular membrane of live bacterial cells with inserted antibodies	Change in Membrane Potential due to binding	1 pg/mL [82]
*Clavibacter*	anion channel formation	Lipid membrane composed of octanethiol, 1,2-Dimyristoyl-sn-glycero-3-phosphocholine, spacerlipid A on a gold electrode which are then coated with phospholipid	Toxic inserted channel proteins were detection by impedance spectroscopy	not given [85]
*Cronobacter* spp.	RNA	Composite sensor composed of glass fiber, cellulose and nitrocellulose membranes with linked oligonucleotide sandwich	Visual colour change due to carbon nanoparticles bound to ssDNA	8 ng or 3 µg/mL [55]
Cucumber mosaic virus	virus particle	Cellular membrane of live fibroblast cells with electroinserted antibodies	Antibody-antigen binding was quantified by the observed change in electric potential	1 ng/mL [84]
	virus particle	Cellular membrane of live mammalian cells with electroinserted antibodies	Antibody-antigen binding was quantified by the observed change in electric potential	1 ng/mL [83]
Cyanobacteria	microcystin MC-LR protein	Ultrabind polyethersulfone membranes with linked protein phosphatase	Microcystin inhibits PP activity, reducing production of yellow pNP from colorless pNPP substrate	0.30 µg/mL [67]
	microcystin MC-RR protein	Ultrabind polyethersulfone membranes with linked protein phosphatase	Microcystin inhibits PP activity, reducing production of yellow pNP from colorless pNPP substrate	0.52 µg/mL [67]
Dengue virus	virus particle	Polyethersulfone membrane with linked DNA capture probe	Reflectometer-based detection of dye-filled liposomes linked to reported probes	serotype 2–50 molecules [65]
	RNA	Nanoporous alumina membrane with linked ssDNA probe	Change in ionic conductivity due to oligonucleotide hybridization in pores was recorded by cyclic voltammetry and DPV	9.55 × 10^−12^ M [40]
	glycoproteins	Lipid membrane modified by Concanavalin A on and gold electrode	Binding of Dengue virus particles was observed using cyclic voltammetry and electrochemical impedance techniques	not given [90]
	RNA	Polyethersulfone membrane with linked DNA capture probe	Reflectometer-based detection of dye-filled liposomes linked to ssDNA reported probes	Roughly 10 PFU/mL [63]
	RNA	Nanomembrane composed of polystyrene-divinylbenzene particles with quaternary ammonium groups and polyamide/polyestertextile fiber embedded in polyethylene with linked oligonucleotide probe	Change in ion current with oligonucleotide hybridization	1 pM [81]
DNA sensing for pathogen detection	DNA	Nanoporous alumina membrane with linked ssDNA probe	EIS-based detection of DNA hybridization in the pores	50 pM [35]
*Enterobacter aerogenes*	whole bacteria	Immunodyne ABC membranes with various linked lectins	Chemometric data analysis of pathogen binding chronocoulometry results were used to distinguish between different pathogens	Not given [80]
*Escherichia coli*	whole bacteria	ImmunodyneABC Nylon membranes coated with 10 different lectins	Detection of pathogen through chronocoulometric results and factor analysis for identification of 4 *E. coli* subspecies.	1.8 × 10^7^ CFU/mL [72]
	whole bacteria	Nylon membrane used to prevent fouling of graphite–Teflon–peroxidase–ferrocene composite electrode	Change in current, due to presence or absence of catalase- based decomposition of hydrogen peroxide, was recorded by the electrode	2 × 10^6^ CFU/mL [69]
	RNA	Nanomembrane composed of polystyrene-divinylbenzene particles with quaternary ammonium groups and polyamide/polyestertextile fiber embedded in polyethylene with linked oligonucleotide probe	Change in ion current with oligonucleotide hybridization	1 pM [81]
whole bacteria	Nanoporous alumina membrane with linked antibodies	Antibody-antigen binding was quantified by impedance amplitude changes	~1000 CFU/mL [39]	whole bacteria
	RNA	Polyethersulfone membrane with linked ssDNA capture probe	Reflectometer-based detection of dye-filled liposomes linked to ssDNA reported probes	5 fmol [66]
	virulence factors	Membranes were composed of either 2,3-di-*O*-phytanylglycerol-1-tetraethylene glycol-d,l-lipoic acid ester lipid, 2,3-di-Ophytanyl-sn-glycerol-1-tetra-ethylene glycol-(3-tryethoxysilane) ether lipid, or cholesterolpentaethyleneglycol and 1,2-di-*O*-phytanoyl-sn-glycero-3 phosphocholine or cholesterol	Bacterial toxins were detected through change in impedance caused by pore formation in the lipid bilayer	not given [51]
	Gold coated PDMS membrane with linked thiols	Stress-based membrane deflection detected by white light and fiber optic interferometers	Distinguish between living and dead cells [44]	whole bacteria
	whole bacteria	Immunodyne ABC membranes with various linked lectins	Chemometric data analysis of pathogen binding chronocoulometry results were used to distinguish between different pathogens	not given [80]
	whole bacteria	Vesicles formed from TRCDA and DMPC	TRCDA vesicles change colour when exposed to lipopolysaccharides from pathogens	~10^8^ CFU [89]
*Escherichia coli* DH1	DNA	Nitrocellulose membranes coated with the contents of lysed *E. coli* cells	PCR was performed and radiolabeled DNA probes were added to bind to the DNA from the lysed cells. The autoradiography was recorded using autoradiography film.	not given [58]
*Escherichia coli* O157:H7	whole bacteria	Nanoporous alumina membrane with linked antibodies	Change in impedance due to antibody-antigen binding was recorded by an electrochemical analyzer	10^2^ CFU/mL [37]
	whole bacteria	Nitrocellulose membrane with linked anti-*E. coli* O157:H7 antibody conjugated to HRP placed over oxygen probe membrane	On pathogen binding, decrease in HRP activity is recorded by a Clark-type oxygen electrode probe	50 cells/mL [57]
	whole bacteria	Polypropylene microfiber membrane coated with conductive polypyrrole and linked with antibodies	Change in resistance due to antibody-antigen binding	log 0–9 CFU/mL [73]
	whole bacteria	Nanoporous nylon membrane with linked antibodies	Pathogen detected by photoluminescent CdSe/ZnS core/shell dendron nanocrystal-conjugated antibodies	2.3 CFU/mL [70]
	whole bacteria	Nylon membrane with linked capture antibody	Sandwich ELISA with NaI, ortho-phenylenediamine and hydrogen peroxide substrates which were measured amperometrically	100 cells/mL [71]
	whole bacteria	Nitrocellulose membrane with linked capture antibody	Sandwich ELISA with luminol-based chemiluminescent output	10^5^–10^6^ CFU/mL [54]
	whole bacteria	Nanofiber nitrocellulose membranes with linked antibodies	Pathogens coated by conductive nanoparticle-conjugated antibodies were immobilized on the membrane and quantified via the change in resistance.	61 CFU/mL [59]
	DNA	Aluminum anodized oxide membrane with linked	Change in ionic conductivity due to DNA hybridization in pores measured by cyclic voltammetry and impedance spectroscopy	0.5 nM [36]
	whole bacteria	Composite sensor composed of glass fiber, cellulose and nitrocellulose membranes with linked capture antibodies	Visual output from sandwich ELISA using 3,3′,5,5′-tetramethylbenzidene and SuperSignal West Femto substrates	1.8 × 10^3^ to 1.8 × 10^8^ CFU/mL [47]
	whole bacteria	Nylon membrane with linked capture antibody	Sandwich ELISA with NaI, ortho-phenylenediamine and hydrogen peroxide substrates which were measured amperometrically	100 cells/mL [71]
	whole bacteria	Nitrocellulose membrane with linked capture antibody	Sandwich ELISA with luminol-based chemiluminescent output	10^5^–10^6^ CFU/mL [54]
	whole bacteria	Nanofiber nitrocellulose membranes with linked antibodies	Pathogens coated by conductive nanoparticle-conjugated antibodies were immobilized on the membrane and quantified via the change in resistance.	61 CFU/mL [59]
	DNA	Aluminum anodized oxide membrane with linked	Change in ionic conductivity due to DNA hybridization in pores measured by cyclic voltammetry and impedance spectroscopy	0.5 nM [36]
	whole bacteria	Composite sensor composed of cellulose and nitrocellulose membranes with linked antibodies	Detection of antigen with conductive nanoparticle-conjugated antibodies and quantification via change in conductance	67 CFU/mL [60]
	whole bacteria	Nanoporous alumina membrane with linked antibodies	Change in ionic impedance of electrolytes in nanopores due to antibody-antigen binding	83.7 CFU/mL [42]
Feline calicivirus	virus particle	Nanoporous polyacrylamide membrane used for pathogen concentration	Antibodies conjugated to fluorescent dye filled liposomes were used to quantify the pathogen	1.6 × 10^5^ PFU/mL [76]
*Giardia lamblia*	*Giardia lamblia* cysts	Gold-coated PCTE membrane filter	Immunogold labeled antigen quantified via Raman spectroscopy	200 cysts/mL [41]
Hepatitis B virus	surface antigen	Nanoporous nylon membrane with linked antibodies	Pathogen detected by photoluminescent CdSe/ZnS core/shell dendron nanocrystal-conjugated antibodies	5 ng/mL [70]
Human Papilloma virus	DNA	Gold membrane with linked bis-peptide nucleic acid probe	Surface acoustic wave based detection of DNA hybridization	1.21 pg/L [43]
Influenza A virus	virus particle	Nitrocellulose membrane coated with antigen	Detection of antigen with magnetic bead-conjugated antibodies which were quantified with a magnetic reader	1 to 250 ng/mL [53]
*Legionella pneumophilla*	DNA	Nanoporous alumina membrane with linked ssDNA probe	Change in ionic conductivity due to oligonucleotide hybridization in pores was recorded by cyclic voltammetry and DPV	3.1 × 10^−13^ M [38]
*Mycobacterium avium* subspecies paratuberculosis	RNA	Polyethersulfone with linked oligonucleotide sandwich	Reflectometer-based detection of dye-filled liposomes linked to reported probes	10 CFU [61]
	whole bacteria	Composite sensor composed of glass fiber, cellulose and nitrocellulose membranes	A primary antibody and secondary conductive nanoparticle-conjugated antibody bind to the antigen, and the change in conductivity is recorded.	serum dilution of 1:80 [48]
*Mycobacterium parafortuitum*	whole bacteria	HPC modified cellulose acetate ultrafiltration membrane with linked antibody	Fluorescently labeled secondary antibodies were used to detect the immobilized pathogen	not given [78]
Potato virus Y	virus particle	Cellular membrane of live mammalian cells with electroinserted antibodies	Antibody-antigen binding was quantified by the observed change in electric potential	minimum detection of 1 ng/mL [83]
*Proteus vulgaris*	whole bacteria	Immunodyne ABC membranes with various linked lectins	Chemometric data analysis of pathogen binding chronocoulometry results were used to distinguish between different pathogens	not given [80]
*Pseudomonas aeruginosa*	DNA	TiO_2_ and TiO_2-_polyethylene glycol membranes on piezoelectric quartz with linked ssDNA probe	DNA hybridization detected by shift in resonant frequency	10^−4^ g/L [45]
	virulence factors	Membranes were composed of either 2,3-di-O-phytanylglycerol-1-tetraethylene glycol-D, l-lipoic acid ester lipid, 2,3-di-Ophytanyl-sn-glycerol-1-tetra-ethylene glycol-(3-tryethoxysilane) ether lipid, or cholesterolpentaethyleneglycol and 1,2-di-O-phytanoyl-sn-glycero-3 phosphocholine or cholesterol	Bacterial toxins were detected through change in impedance caused by pore formation in the lipid bilayer	not given [51]
*Saccharomyces cerevisiae*	whole bacteria	Immunodyne ABC membranes with various linked lectins	Chemometric data analysis of pathogen binding chronocoulometry results were used to distinguish between different pathogens	not given [80]
*Salmonella Newport*	GIII bacteriophage	Polypyrrole modified microporous polycarbonate membrane	Pathogen cells drawn into membrane pores, GIII bacteriophage added to pathogen and change in impedance recorded	not given [79]
*Salmonella spp.*	whole bacteria	Nitrocellulose membrane with linked capture antibody	Sandwich ELISA with luminol-based chemiluminescent output	10^6^–10^7^ CFU/mL [54]
*Salmonella Typhi*	whole bacteria	Polycarbonate membranes with linked antibodies	Sandwich ELISA with colourimetric output from 3,3',5,5' tetramethyl benzidine-hydrogen peroxide substrates	2 × 10^3^ cells/mL [75]
*Salmonella typhimurium*	whole bacteria	Vesicles formed from TRCDA and DMPC	TRCDA vesicles change colour when exposed to lipolysaccharides from pathogens	~10^8^ CFU [89]
	whole bacteria	Nitrocellulose membrane coated with	Urease, linked to bacteria on the surface, converts urea to ammonia and CO_2_ which results in a pH change which is measured as a change in electric potential	119 CFU [52]
	whole bacteria	Composite sensor composed of glass fiber, cellulose and nitrocellulose membranes with linked capture antibodies	Visual output from sandwich ELISA using chemiluminescent substrate solution quantified by CMOS image sensor	4.22 × 10^3^ CFU/mL and 1.1 × 10^2^ CFU/mL with pre-separation and concentration [49]
*Shigella sonei*	whole bacteria	Vesicles formed from TRCDA and DMPC	TRCDA vesicles change colour when exposed to lipolysaccharides from pathogens	~10^8^ CFU [89]
*Stapholococcus aureus*	whole bacteria	Polyethersulfone membrane	Pathogen cells were labeled with HRP conjugated antibodies, collected by the membrane and quantified by a luminol-based luminescent reaction	3.8 × 10^4^ CFU/mL [68]
	DNA (enterotoxins B gene)	Membranes composed of egg phosphatidylcholine, cholesterol and hexadecylamine with linked ssDNA probes	DNA hybridization detected by change in current through the membrane	20 ng/mL [87]
	whole bacteria	Immunodyne ABC membranes with various linked lectins	Chemometric data analysis of pathogen binding chronocoulometry results were used to distinguish between different pathogens	not given [80]
	virulence factors	Membranes were composed of either 2,3-di-O-phytanylglycerol-1-tetraethylene glycol-D,L-lipoic acid ester lipid, 2,3-di-Ophytanyl-sn-glycerol-1-tetra-ethylene glycol-(3-tryethoxysilane) ether lipid, or cholesterolpentaethyleneglycol and 1,2-di-O-phytanoyl-sn-glycero-3 phosphocholine or cholesterol	Bacterial toxins were detected through change in impedance caused by pore formation in the lipid bilayer	~240 pM [51]
	whole bacteria	Nanoporous alumina membrane with linked antibodies	Antibody-antigen binding was quantified by impedance amplitude changes	~1000 CFU/mL [39]
	whole bacteria	Celluloseacetate membrane filters	Pathogen-antibody/gold nanoparticle/magnetic nanoparticle complexes were filtered through the membrane and the colour change was quantified by the optical density.	1.5 × 10^3^ CFU for pure bacteria and 1.5 × 10^5^ CFU in milk [77]
	whole bacteria	Nanoporous alumina membrane with linked antibodies	Change in impedance due to antibody-antigen binding was recorded by an electrochemical analyzer	10^2^ CFU/mL [37]
*Streptococcus pneumoniae*	whole bacteria	Nylon membrane used to prevent fouling of graphite–Teflon–peroxidase–ferrocene composite electrode	Change in current, due to presence or absence of catalase- based decomposition of hydrogen peroxide, was recorded by the electrode	2 × 10^5^ cfu/mL [69]
Tobacco mosaic virus	virus particle	Cellular membrane of live bacterial cells with electroinserted antibodies	Antibody-antigen binding was quantified by the observed change in electric potential	1 pg/mL [82]
Tobacco rattle virus	virus particle	Cellular membrane of live mammalian cells with electroinserted antibodies	Antibody-antigen binding was quantified by the observed change in electric potential	1 ng/mL [83]
*Vibrio cholerae*	DNA	Composite sensor composed of glass fiber and cellulose membranes with linked oligonucleotide sandwich	Visual colour change due to gold nanoparticles bound to ssDNA	5 ng or 250 ng/mL [46]
	cholera toxin protein complex	Lipid membrane composed of octanethiol on a gold electrode which was then coated with DPPC and GM1	Cholera toxin induced liposome agglutination on the piezoelectric sensor was detected by the resonant frequency shift	25 ng/mL [86]
	cholera toxin protein complex	Polydiacetylene liposomes with incorporated ganglioside, GM1	Cholera toxin induces a change in the liposome light absorption	not given [51]
*Yersinia pestis*	whole bacteria	Composite sensor composed of glass fiber, cellulose and nitrocellulose membranes with linked capture antibodies	Secondary antibodies conjugated to up-converting phosphor particles were excited, and the resultant luminescence was quantified by a photomultiplier tube	10^4^ CFU/mL [50]

## 7. Conclusions

Membranes have been used in a number of biosensor designs for the detection of pathogens. In this review, the central components of these sensors—membrane composition, detection biomolecule, linking process, transduction process and sensitivity—were divided up and examined individually. For the majority of membrane biosensors, the membrane material is the most important aspect for the sensing application, largely for functionalization purposes. In cases where the membrane is used for filtration or is an integral part of the detection or transduction process, other properties of the membranes become more important. A number of reports involve the use of aluminum oxide. It was selected for properties such as high resistivity, well defined small pore size, high pore density and the simplicity with which it can be functionalized. Other inorganic materials were mainly used in conjunction with organic materials, sometimes to facilitate linking processes such as sulfide-gold linkages, and sometimes to enhance transduction such as surface-enhanced Raman spectroscopy. Glass membranes were used for capillary action to combine organic molecules used in the sensor while silicon nitride was used for mechanical support.

Several more reports however involved organic membranes rather than inorganic ones. Predictably, the most common membrane material is nitrocellulose, which is commonly used in biomedical applications. Other common membrane materials were polyethersulphone and nylon. Several researchers acquired readily available commercial membranes, many of which are prefunctionalized for adhesion or covalent bonding. Use of lipid-bilayer based membranes was also reported.

Antibodies and nucleic acid hybridization were by far the most common methods used for pathogen detection. Antibodies are often used in sandwich applications, with a capture antibody for initial detection and a conjugated antibody for transduction. Nucleic acid-based detection mostly consisted of simple hybridization of complementary probe and DNA or RNA strands from pathogens. Other approaches included bacteriophage-mediated detection and non-antibody based protein-protein interactions.

Linking procedures are frequently used to attach sensing/detection molecules to the membranes. Covalent bonding is often preferable as it strongly secures the detection molecules to the surface, preventing them from being washed off. The most common covalent crosslinker used was glutaraldehyde. EDC and Sulfo-NHS were also common for covalent crosslinking. Silanes, which bind covalently to a variety of surfaces, were used to either directly link molecules to membrane surfaces or indirectly through a crosslinker like glutaraldehyde. Simple adsorption to the membrane surface without a linker was also used, particularly for prefunctionalized membranes. Streptavidin-biotin binding may be used to augment this process by orienting the sensing molecule being linked to the surface. In the alternative, electroporation has also been employed for attaching molecular probes.

Electrical and optical transduction methods were most commonly used to convert biological sensing into a readable output. In many cases, the act of detecting the nucleic acids (through hybridization) or protein binding sufficiently changed the electrical properties of the membranes (such as resistance, voltage, current, impedance) that they could be observed via simple electrodes. In some cases electrical current was generated through enzymatic or other chemical reactions. In other approaches, the change in electrical current in porous membranes due to nucleic acid or protein binding in the pores was recorded. Some bacteria produce proteins which create pores in membranes.

In turn, optical transduction methods can be categorized based on type of output, including color changes, light emission, and spectroscopic/interferometric approaches. Colour change was achieved in a number of ways, including dye-containing liposomes linked to nucleic acid tags, antibodies conjugated to gold and carbon nanoparticles, several horseradish peroxidase-catalyzed and other chemical reactions, and change of colour due to protein interaction with liposomes. Some similar methods were employed for transduction through photon emission.

Other transduction involved mass sensing through leaky surface acoustic waves, piezoelectric effects, magnetic readout of antibodies conjugated to magnetic beads, as well as oxygen metering of the cellular respiration of living bacterial cells.

The main goal of a pathogen biosensor is to specifically detect as few live bacteria or infectious virus particles in as large a volume as possible. Viruses and bacteria are mainly detected as whole units, through their structural proteins, through the proteins or other materials they produce, or through their DNA or RNA sequences. For certain proteins, such as those found inside the pathogen, and for DNA or RNA sequences, this may involve additional processing to break up the pathogen and release the DNA/RNA or protein being detected. Unfortunately, it can be very difficult to compare the effectiveness of nucleic acid sensors, with protein sensors and whole virus or bacterial sensors. A whole virus or bacterium is naturally much more massive than a protein, and a protein is more massive than a small strand of DNA or RNA. Therefore, nucleic acid detection methods generally need to have smaller mass/volume detection limit than protein detection methods or whole pathogen detection methods.
